# Newly Emerging Feeding Difficulties in a 33-Year-Old Adult With CHARGE Syndrome

**DOI:** 10.14740/jocmr2288w

**Published:** 2015-12-03

**Authors:** Alexandra Hudson, Kim Blake

**Affiliations:** a Department of Medicine, Dalhousie University, Canada; b Department of Pediatrics, Faculty of Medicine, Dalhousie University, Canada

**Keywords:** CHARGE syndrome, Feeding difficulties, Mouth over-stuffing

## Abstract

Feeding and swallowing difficulties are common among individuals with CHARGE syndrome. Many children require gastrostomy tube feeding in their early years and often undergo a delay in feeding and oral-motor skill development. There is little information available on adults with CHARGE syndrome, and the feeding difficulties they face. The present case describes newly emerging mouth over-stuffing feeding behaviors and feeding difficulties in a 33-year-old adult with CHARGE syndrome who had not undergone feeding therapy since childhood.

## Introduction

CHARGE syndrome is a genetic disorder caused by a mutation in the CHD7 gene on chromosome 8q12.1, and is prevalent in approximately 1 in 15,000 - 17,000 births [1]. The syndrome involves a cluster of physical symptoms and features that have variability among individuals. Each letter in the word CHARGE stands for a common feature of the syndrome: coloboma of the eye, heart defects, atresia of the choanae, retardation of growth and development, genitourinary defects, and ear abnormalities [1]. The most cardinal physical features can also be described as the four C’s: coloboma, choanal atresia/stenosis, characteristic ear anomalies, and cranial nerve dysfunction [2]. CHARGE syndrome is most often initially clinically diagnosed and subsequently confirmed by genetic testing [1-3].

Feeding difficulties are prevalent in CHARGE syndrome, and up to 90% of children with CHARGE syndrome have been reported to receive tube feeding at some point in their life [4]. Feeding difficulties are thought to be due to the cranial nerve dysfunction, although there has been no correlation between a single clinical characteristic of CHARGE syndrome and severe feeding difficulties [2, 4, 5]. Common clinical characteristics in CHARGE syndrome such as weak sucking, dysfunctional swallowing and chewing, aspiration, and gastroesophageal reflux may also account for feeding difficulties experienced [4]. The Canadian Pediatric Surveillance Program identified severe feeding difficulties as a significant cause of morbidity in CHARGE syndrome, as 90% of the deceased patients identified in Canada were at risk for aspiration or choking due to gastroesophageal reflux, chewing, and swallowing difficulties [6]. Death due to choking during eating has also been described in a child with CHARGE syndrome [7]. In addition, reflux, breathing difficulties, and feeding difficulties were found to be associated with decreased survival in childhood [7]. Therefore, identification and management of feeding difficulties in CHARGE syndrome is paramount.

Evidence surrounding the clinical management of CHARGE syndrome in adulthood is scarce. Furthermore, most of the information concerning feeding difficulties in CHARGE syndrome is discussed in the context of infancy and childhood [4, 8]. Individuals with CHARGE syndrome however, now have an increased life expectancy due to improved management of their complex condition [6]. Despite new medical and behavioral issues that have now been reported to emerge in adolescence and adulthood in CHARGE syndrome [9, 10], to our knowledge, no reports of newly developed problematic feeding behaviors in an adult with CHARGE syndrome exist to date.

We herein report a case of newly developed mouth over-stuffing feeding behavior and feeding difficulties in a 33-year-old male with CHARGE syndrome.

## Case Report

A 33-year-old Caucasian male with CHARGE syndrome presented with new problematic feeding behaviors that included mouth over-stuffing while eating. He had a genetic diagnosis of CHARGE syndrome since the age of 12 years. His major CHARGE syndrome characteristics included bilateral coloboma of the retina and optic nerve, and abnormalities of the external, middle, and inner ear. He had cranial nerve dysfunction, which manifested in hearing and swallowing problems, but did not have facial palsy. He had heart defects that included tetralogy of Fallot and a ventral septal defect, as well as a cleft lip and cleft palate. Additional CHARGE syndrome characteristics included genital hypoplasia, growth deficiency, developmental delay, and distinct CHARGE syndrome facial features (Table 1 [2, 3, 11] for summary). Furthermore, he experienced blindness, deafness, and was non-verbal.

**Table 1:** Phenotypic Features of CHARGE Syndrome

	Present case	Frequency [11]
Major criteria [2, 3]		
Coloboma	Yes	75-90%
Choanal atresia/stenosis	No	65%
Hypoplasia of semicircular canals	Not tested	90%
Minor criteria [2, 3]		
Cranial nerve anomalies (hearing, swallowing problems)	Yes	50-90%
Growth deficiency	Yes	70-80%
Malformation of the internal/external ear	Yes	90-100%
Cardiovascular abnormalities	Yes	50-85%
Developmental delay	Yes	70%
Cleft lip/palate	Yes	15-20%
Genital hypoplasia	Yes	50-70%
Distinct CHARGE facial features^a^	Yes	N/A

^a^Include a square face, flat mid-face, and prominent forehead [11].

Unlike many individuals with CHARGE syndrome, he was never tube-fed as a child. As an infant, he experienced an oral aversion towards oral feeding and had a very sensitive mouth. His early bottle feeding as an infant required a bottle with a wider nipple opening and a thickening agent to help him control the flow of the formula. He had previously used modified feeding utensils, which included a modified bowl with a lip on it to aid in scooping food onto his utensils, but as an adult he was no longer using any specialized or modified utensils. Although he still needed assistance in cutting his food, he was able to feed himself. Nonetheless, he required intensive supervision during meals and was never left unattended while eating. He did not have any feeding issues with puree, mashed, or chewable/solid food textures, but had persistent difficulties with liquids. For example, he would drink a whole cup of water at once if given the means. The only food textures that he did not tolerate were hard vegetables and fruit. He experienced swallowing difficulties as an infant but did not experience any swallowing difficulties in his adulthood.

This 33-year-old male would also periodically hold saliva in his mouth without swallowing, which required a physical prompt (stroking his neck) from his caregiver as a reminder to swallow. Chewing was another major feeding difficulty for him. His caregiver also reported that this individual with CHARGE syndrome would frequently store food in his cheeks, letting it soften sufficiently for him to swallow it. Any chewable/solid texture food (i.e. meat) had to be cut into very tiny pieces for him to chew and eat.

Previous feeding interventions included occupational therapy from age 3 to 7 years old. Occupational therapists worked on improving his ability to feed himself and to develop a technique for eating, in particular, pureed textures. He never received any other feeding therapy and had no surgical history for the management of swallowing or feeding. He experienced gastroesophageal reflux at age 30 years, which was well managed by Prilosec (omeprazole) and discontinued after 6 months. Although he was no longer taking Prilosec, he currently would avoid lying flat after finishing a meal and periodically took sodium bicarbonate to prevent and treat any gastroesophageal reflux symptoms.

Mouth over-stuffing feeding behavior emerged at the age of 28 years and was seemingly sporadic. His parent described this feeding behavior as “continuously putting food in his mouth”. While living at a group home, the staff had to closely supervise his eating and had to continuously monitor and limit the amount of food given to him at a single time. Staff and caregivers carefully developed a physical prompt (putting his hand down during meals) as a reminder for him to not over-stuff his mouth. His parent reported that this was successfully implemented. A liquid chaser (i.e. water) was never introduced to reduce over-stuffing as he had a very specific order of completing his meals (drinking after the meal had finished). Supervision while eating also prevented him from storing food in his cheeks while eating, a feeding behavior that he had also newly developed since the age of 28 years. Furthermore, close supervision while eating prevented any choking episodes.

Close supervision while eating, limiting the amount of food available on his plate, and using a behavioral intervention (a physical prompt) were successful in reducing this young man’s problematic mouth over-stuffing feeding behavior and preventing consequences such as choking.

## Discussion

Feeding difficulties are prevalent in CHARGE syndrome, with the majority of children needing to be tube fed before oral feeding [4]. Difficulties are thought to be due to cranial nerve dysfunction, which can cause problems such as a weak swallowing and chewing, gastroesophageal reflux, and aspiration [4]. The individual described in this case report demonstrated cranial nerve dysfunction, which resulted in difficulties with hearing, swallowing, chewing, and gastroesohpageal reflux. Cranial nerves that are hypothesized to contribute to feeding difficulties in CHARGE syndrome include the trigeminal nerve (cranial nerve V), which is responsible for the innervation of the muscles involved in chewing, as well as relaying sensory information from the face [5, 12]. The glossopharyngeal nerve (cranial nerve IX) innervation of the pharynx and mediation of the swallowing reflex, and the vagus nerve (cranial nerve X) innervation of the pharynx and larynx, are also thought to be important contributors to feeding difficulties when dysfunctional [5]. This young man’s cranial nerve dysfunction adds to the hypothesis of cranial nerve involvement in feeding difficulties seen in CHARGE syndrome such as mouth over-stuffing during feeding.

There is very little information regarding CHARGE syndrome in adulthood and the issues they face. One study revealed that many of the individuals faced new medical issues in this age range, but new feeding issues were not mentioned [9]. This study further identified a high number of reported behavioral concerns in adolescents and adults with CHARGE syndrome [9]. A case report of a 33-year-old individual with CHARGE syndrome demonstrated adulthood issues that included gallstones, delayed puberty, osteoporosis, behavioral disturbances and anxiety, but did not report any adult feeding difficulties [10]. One study assessed nutrition and bone health in adolescents (> 13 years old) and adults with CHARGE syndrome and found that over half of the individuals had previously had feeding difficulties in childhood; however, there was no assessment of adult-onset feeding difficulties [13].

In conclusion, we present a unique case of an adult with CHARGE syndrome who had new onset feeding difficulties that developed late in his life. The present case report adds to the limited available literature on adults with CHARGE syndrome and the resulting feeding issues they face. Clinicians should be aware that individuals with CHARGE syndrome could experience feeding difficulties at any point in their life, and those who do not need G-tube feeding as a child may still later experience feeding difficulties that require intervention.
